# Executive function mediates prefrontal excitation–inhibition balance and emotion recognition in euthymic bipolar disorder

**DOI:** 10.1017/S0033291725102018

**Published:** 2025-10-10

**Authors:** Cheng Ying Wu, Chih-Yu Chang, Shyh-Yuh Wei, Hui Hua Chang, Ying Tsung Tsai, Tsung-Hua Lu, Ren-Yi Lin, Yen Kuang Yang, Po See Chen, Yu-Lien Huang, Huai-Hsuan Tseng

**Affiliations:** 1 Department of Psychiatry, National Cheng Kung University Hospital, College of Medicine, National Cheng Kung University, Tainan, Taiwan; 2 Department of Medicine, College of Medicine, National Cheng Kung University, Tainan, Taiwan; 3 Institute of Clinical Pharmacy and Pharmaceutical Sciences, National Cheng Kung University, Tainan, Taiwan; 4 School of Pharmacy, College of Medicine, National Cheng Kung University, Tainan, Taiwan; 5 Department of Pharmacy, National Cheng Kung University Hospital, College of Medicine, National Cheng Kung University, Tainan, Taiwan; 6 Department of Pharmacy, National Cheng Kung University Hospital, Dou-Liou Branch, Yunlin, Taiwan; 7 Mind Research and Imaging Center, National Cheng Kung University, Tainan, Taiwan; 8 Department of Psychology, National Cheng Kung University, Tainan, Taiwan; 9 Institute of Behavioral Medicine, College of Medicine, National Cheng Kung University, Tainan, Taiwan; 10 Department of Psychology, Chung Shan Medical University, Taichung, Taiwan

**Keywords:** ^1^H-MRS, bipolar disorder, E/I imbalance, GABAergic, glutamatergic, social cognition

## Abstract

**Background:**

Euthymic bipolar disorder (euBD) patients exhibit deficits in neurocognitive and social cognitive functioning compared to healthy controls (HCs). Our prior research has shown that the excitatory/inhibitory (*E*/*I*) imbalance in the default mode network (DMN) is linked to executive function in euBD. Neurocognitive impairments are associated with social cognition deficits in individuals with mental disorders. Given this connection, this study posits *E*/*I* imbalance within the DMN is associated with social cognition, with executive function as a mediator.

**Methods:**

Seventy-five HCs and 49 euBD individuals were recruited. Using the emotion recognition task, Diagnostic Analysis of Nonverbal Accuracy 2-Taiwan version (DANVA-2-TW) and cognitive flexibility task, Wisconsin Card Sorting Test (WCST), we assessed emotion recognition and prefrontal function. Proton magnetic resonance spectroscopy (^1^H-MRS) measured metabolites in the posterior cingulate cortex (PCC) and medial prefrontal cortex/anterior cingulate cortex (mPFC/ACC), quantifying excitatory glutamate+glutamine (Glx) and inhibitory GABA to calculate the E/I ratio.

**Results:**

euBD patients showed poorer emotion recognition (*p* = 0.020) and poorer cognitive flexibility (fewer WCST categories completed, *p* = 0.002). A negative association was found between emotion recognition and the E/I ratio in the mPFC/ACC of the BD patients (*r* = −0.30, *p* = 0.034), which was significantly mediated by cognitive flexibility (*Z* = −2.657, *p* = 0.007).

**Conclusion:**

The BD patients demonstrate deficits in emotion recognition, linked to an altered E/I balance in the prefrontal cortex, and the cognitive flexibility, a key aspect of executive function, mediates the impact of the E/I ratio on emotion recognition accuracy in euBD patients.

## Introduction

Social cognition refers to cognitive processes, including social cue perception, inferring others’ thoughts or emotions, and managing emotional reactions or behavior to others (Arioli, Crespi, & Canessa, 2018). This ability is evolutionarily advantageous for individuals who live in groups, as it allows for enhanced protection against predators (Tomasello, 2014). Social cognitive function is impaired in individuals with Bipolar disorder (BD), who may take longer to differentiate emotions, and exhibit lower accuracy compared to healthy controls (HCs), particularly when under time pressure (Gillissie et al., 2022). For these individuals, their mood swings can further impair their ability to accurately interpret social cues and respond appropriately, leading to a vicious cycle of increasingly impaired social cognitive difficulties (Forgas, Bower, & Krantz, 1984). We have previously demonstrated that individuals with BD exhibit social cognitive deficits, particularly in emotion recognition, across multiple studies (Chang et al., 2024; Lee et al., 2022; Tsai et al., 2023a; Tsai et al., 2023b). These deficits are closely linked to broader cognitive impairments (Tsai et al., 2023b) and contribute to less favorable real-world outcomes, including interpersonal function (Lee et al., 2022) and quality of life (Tamura et al., 2022). Social cognition serves as a critical bridge between cognitive deficits and functional outcomes (Ospina et al., 2018), and can be an important intervention target to enhance the quality of life for individuals with BD (Gillissie et al., 2022).

Social cognition impairments in BD are underpinned by both neurofunctional and neurochemical changes in the brain. Several neurotransmitters, such as glutamate and GABA, and specific regions, such as the default mode network (DMN), salience network, and prefrontal cortex (PFC), have been implicated (Lopatina et al., 2018). The PFC is a crucial regulator of social cognition, and disturbances in prefrontal microcircuitry contribute significantly to the pathophysiology of social deficits in psychiatric disorders. (Bicks, Koike, Akbarian, & Morishita, 2015; Tso et al., 2021). While mPFC is one of the critical hubs of the DMN, the manifestation of DMN in social cognitive deficits is also evident (Tso et al., 2021).

A direct link between an impaired balance of excitatory and inhibitory neurotransmitters and changes in social behavior has been confirmed in recent studies in mice and humans (Lopatina et al., 2018). The relationship between *E*/*I* balance in the PFC and social behavior was further supported by evidence showing that modulating the *E*/*I* balance in the medial prefrontal cortex (mPFC) of mice can rescue autism-like social behavior deficits (Selimbeyoglu et al., 2017). In BD, previous research has identified excitatory (glutamate and glutamine) and inhibitory (GABA) neurometabolite alterations and their equilibrium by Proton magnetic resonance spectroscopy (^1^H-MRS) in mPFC/ACC (Scotti-Muzzi, Umla-Runge, & Soeiro-de-Souza, 2021; Sosa-Moscoso et al., 2022) and PCC/PCu, the hub of the DMN (Tseng et al., 2024).

Furthermore, studies have demonstrated a significant positive association between GABA and DMN deactivation; DMN deactivation was also correlated with working memory, suggesting functional relevance to its deactivation (Hu, Chen, Gu, & Yang, 2013). Our research team has further identified an association between *E*/*I* imbalance within the DMN and executive function performance in individuals with euBD (Tseng et al., 2024). Furthermore, evidence suggests that neurocognitive impairments, particularly in executive function and memory, are closely linked to deficits in social cognition among individuals with severe mental disorders, including BD (Lancaster, Evans, Bond, & Lysaker, 2003).

In light of the above findings, we hypothesize that the *E*/*I* ratio in the mPFC or PCC—key hubs of the DMN—is associated with impaired emotion recognition in individuals with euBD. Furthermore, we examine whether neurocognitive function mediates this association, providing insight into the potential pathway by which neurochemical imbalance impacts social cognitive performance.

## Methods

### Ethics statement

The study received approval from the Institutional Review Board for the Protection of Human Subjects at National Cheng Kung University Hospital. Patients were recruited from the hospital’s psychiatric outpatient department, while healthy volunteers were enlisted via online platforms and public advertisements. All participants provided written informed consent before enrollment.

### Participants

Our study enrolled 49 euBD patients according to the *Diagnostic Systematic Manual-5th edition.* The BD patients were recruited from outpatient psychiatry clinics in the Department of Psychiatry, National Cheng Kung University Hospital, College of Medicine. Seventy-five HCs were enrolled from the community through advertisement. All participants, aged 18–65 years, underwent assessment by an attending psychiatrist using the Chinese adaptation of the Mini International Neuropsychiatric Interview (Sheehan et al., 1998). The severity of depressive and manic symptoms was quantified with the 17-item Hamilton Depression Rating Scale (HDRS) and the 11-item Young Mania Rating Scale (YMRS). Euthymia was defined as achieving scores of 7 or lower on both the HDRS and YMRS scales (Zhang et al., 2018). The exclusion criteria were as follows: (i) a serious surgical condition or physical illness; (ii) major mental illnesses except BD for the BD patients; (iii) pregnancy or breastfeeding; (iv) substance abuse within the past 3 months, except tobacco use disorder; (v) previous use of any psychotropic agent in the HCs; and (vi) an organic mental disease, mental retardation, or dementia. This cohort partly overlaps with that described in our previously published work (Tseng et al., 2024).

### Image parameters: proton magnetic resonance spectroscopy (^1^H-MRS)

Participants received imaging at the Mind Research and Imaging Center, National Cheng Kung University, utilizing a 3.0 Tesla MRI scanner (GE Discovery MR750, GE Medical Systems) with an 8-channel head coil for data acquisition. ^1^H-MRS was employed to evaluate excitatory (glutamate + glutamine, Glx) and inhibitory (GABA) neurometabolites, enabling the computation of E/I ratios. Glx levels were quantified via point-resolved spectroscopy (PRESS), while GABA concentrations were examined with MEGA-PRESS. The regions of interest, including the mPFC/ACC and PCC/PCu, were selected based on predefined anatomical coordinates (Hu et al., 2013; Kegeles et al., 2012). Neurometabolites were quantified using LCModel, with a Cramér-Rao lower bound (CRLB) threshold of <20%. Partial volume correction accounted for tissue composition using segmentation and analysis tools (Gannet and MRSParVolCorr). This methodology has been previously described in our team’s prior publication (Tseng et al., 2024).

### Social cognition and neuropsychological assessment

#### Non-verbal emotion recognition (DANVA-2-TW)

Emotional expressions, while partly universal, vary subtly across cultures, affecting the accuracy of nonverbal emotion recognition based on race, culture, and gender (Jack, Garrod, Yu, Caldara, & Schyns, 2012; Wickline, Bailey, & Nowicki, 2009). To measure this accuracy in Han Chinese, we used the culturally adapted Diagnostic Analyses of Nonverbal Accuracy 2, Taiwanese Version (DANVA-2-TW). This version, based on the original DANVA-2 (Nowicki & Duke, 1994), has been applied in Taiwanese clinical studies (Pan, Tseng, & Liu, 2013). The DANVA-2-TW includes 60 facial photos and 60 voice recordings depicting happiness, sadness, anger, fear, and neutral emotions, with 12 examples per emotion. Photos were shown on a 1024 × 768 screen, and audio was played via headphones. Accuracy was calculated as the proportion of correct responses per (Pan, Tseng, & Liu, 2013) emotion, ranging from 0 (completely inaccurate) to 1 (completely accurate).

#### Wisconsin Card-Sorting Test (WCST)

An experienced clinical neuropsychologist administered the WCST, which consisted of 64 cards. All index definitions followed the guidelines outlined in the WCST manual (Heaton, Chelune, Talley, Kay, & Curtiss, 1993). Participants completed a computerized version of the WCST, where they matched response cards to four stimulus cards based on color, shape, or quantity, receiving feedback after each attempt. The patients were not informed about the dimensions beforehand. Once patients successfully sorted 10 cards within a given category, they were instructed to reclassify them based on a new category. Performance on the WCST was evaluated using the number of completed categories and perseverative errors.

### Statistical analysis

Chi-squared test was used to compare the demographic and clinical characteristics of the participant groups for dichotomous variables. Differences between the BD and HC groups were assessed through independent *t*-tests. Likewise, MRS variables were examined using the same method, with additional adjustments for age and sex performed via analysis of covariance (ANCOVA) to account for potential confounding factors.

To explore the relationship between emotion recognition and the *E*/*I* ratio in BD, Pearson correlation analysis was performed to assess the association between MRS variables and DANVA-2-TW within the BD group. A significance threshold of *p* < 0.05 was applied to all statistical tests. Mediation effects were evaluated using the Sobel Test Calculator. All statistical analyses were conducted using IBM SPSS Statistics, version 17 (IBM Corporation, Armonk, NY, USA).

## Results

### Demographic data and group differences

We compared the BD and HC groups in terms of age, sex, educational years, clinical mood symptoms, emotion recognition ability, neurocognition, and neurometabolite variables (Table 1). The BD group had a higher proportion of females (*p* = 0.001) and was on average older (38.31 ± 13.22, *p <* 0.001) than the HC group. Hence, age and sex were controlled when comparing emotion recognition ability, neurocognition, and neurometabolite variables between the BD and HC groups. The BD group had higher subsyndromal depressive and manic symptoms compared to the HC group, as measured by the HDRS (*p* = 0.014) and YMRS (*p* < 0.001) scales, respectively.

**Table 1.** Demographic and clinical data

	Healthy controls (*N* = 75)		Bipolar disorder (*N* = 49)		Statistics	
	Mean	± SD	Mean	± SD	t/X^2^	*p*
Gender (M/F)	51/	24	18/	31	11.74	0.001
Age, yr	30.88	±8.36	38.31	±13.22	−3.84	<0.001
Educational years	16.39	±2.17	14.96	±2.01	3.64	<0.001
Young mania rating scale	0.19	±0.65	1.31	±1.65	−5.29	<0.001
Hamilton depression rating scale	1.19	±1.31	1.98	±2.22	−2.50	0.014

As shown in Table 2, the BD group had lower abilities in recognizing happy (0.73 ± 0.16, *p* = 0.002), sad (0.57 ± 0.19, *p* = 0.021), angry (0.55 ± 0.19, *p* = 0.036) and overall emotion (0.60 ± 0.19, *p* = 0.020) compared to the HC group. Moreover, the BD patients performed worse in the WCST categories completed test than the HCs (2.73 ± 1.69, *p* = 0.002) but not in perseverative error (11.20 ± 10.85, *p* = 0.433).

**Table 2.** Neuroimaging and neuropsychological variables

	Healthy controls			Bipolar disorder			Statistics			
	*N*	Mean	± SD	*N*	Mean	± SD	*t*	*p*	*F* ^a^	*p* ^a^
MRS (without correction)										
Glx in PCC	68	9.17	±0.65	47	9.60	±0.94	−2.92	0.004	11.98	0.001
CRLB(range)		4.91	±0.29 (4–5)		4.74	±0.53 (4–6)				
Glx in mPFC/ACC	75	8.50	±2.36	49	9.91	±2.94	−2.95	0.004	6.62	0.011
CRLB(range)		9.56	±2.86 (6–19)		8.82	±2.49 (5–17)				
GABA in PCC	68	2.00	±0.31	48	1.98	±0.27	0.26	0.797	0.02	0.900
CRLB(range)		5.17	±1.45 (3–9)		5.04	±1.40 (4–12)				
GABA in mPFC/ACC	75	1.78	±0.50	49	1.81	±0.46	−0.31	0.755	0.19	0.660
CRLB(range)		8.15	±3.30 (4–20)		8.57	±2.96 (5–19)				
Glx/GABA ratio										
in PCC	68	4.70	±0.75	47	4.91	±0.76	−1.48	0.141	2.69	0.104
in mPFC/ACC	75	5.09	±1.82	49	5.88	±2.43	−2.05	0.042	2.77	0.099
DANVA–2-TW										
Happy	75	0.83	±0.10	49	0.73	±0.16	4.00	<0.001	10.55	0.002
Sad	75	0.66	±0.13	49	0.57	±0.19	3.35	0.001	5.47	0.021
Angry	75	0.65	±0.14	49	0.55	±0.19	3.45	0.001	4.49	0.036
Fearful	75	0.67	±0.16	49	0.57	±0.24	2.80	0.006	0.90	0.346
Total	75	0.70	±0.11	49	0.60	±0.19	3.70	<0.001	5.54	0.020
WCST										
Perseverative error	75	8.32	±5.82	49	11.20	±10.85	−1.92	0.057	0.62	0.433
Categories completed	75	3.81	±1.16	49	2.73	±1.69	4.21	<0.001	9.56	0.002

Abbreviations: MRS, magnetic resonance spectroscopy; Glx: glutamate + glutamine signal complex; PCC: posterior cingulate cortex; mPFC/ACC: medial prefrontal cortex/anterior cingulate cortex; GABA: gamma-aminobutyric acid; CRLB: Cramér-Rao lower bound; DANVA-2-TW: diagnostic analyses of nonverbal accuracy, Taiwanese version.

^a^ Controlling for age and sex.

With regard to MRS variables, the BD patients showed higher glutamate complex levels in both of the PCC (9.60 ± 0.94, *p* = 0.001) and mPFC/ACC (9.91 ± 2.94, *p* = 0.011). The *E*/*I* ratio was higher in mPFC/ACC and remained a trend of significance after controlling for age and sex (*p* = 0.099).

### The association of the E/I ratio and emotion recognition

**Figure 1.**
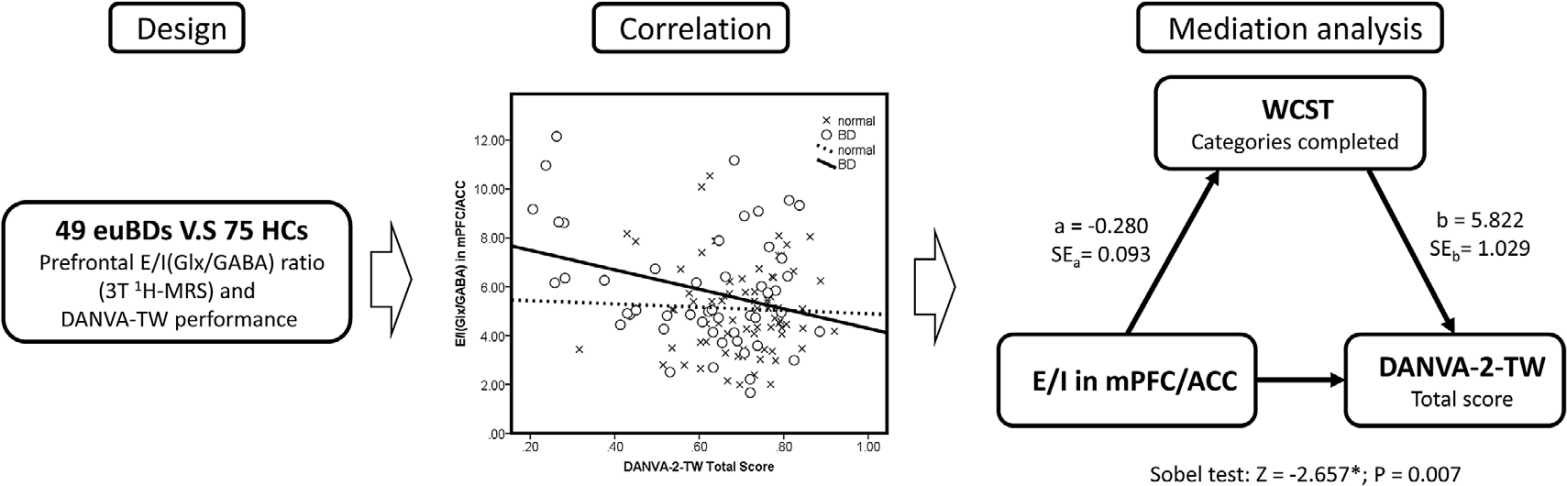
Summary of study design, correlation, and mediation analysis. *Note:* euBD patients (*n* = 49) and HCs (*n* = 75) underwent 3 T ^1^H-MRS and emotion recognition assessment (DANVA-2-TW). In BD, a negative correlation was found between prefrontal *E*/*I* ratio and emotion recognition. Mediation analysis showed WCST performance partially mediated this association (Sobel *Z* = −2.657, *p* = 0.007). Abbreviations: euBD, euthymic bipolar disorder; HCs, healthy controls; 1H-MRS, Proton magnetic resonance spectroscopy; DANVA-2-TW, Diagnostic Analysis of Nonverbal Accuracy 2-Taiwan version; *E*/*I*, excitatory/inhibitory; WCST, Wisconsin Card Sorting Test; mPFC/ACC, medial prefrontal cortex/anterior cingulate cortex.

A negative association between emotion recognition (DANVA-2-TW total score) and the glutamatergic-GABAergic balance was observed in the mPFC/ACC of the BD patients (*r* = −0.30, *p* = 0.034) (Figure 1). However, this relationship was not seen in the HC group (*r* = −0.04, *p* = 0.732).

After including WCST categories completed as a control variable in the correlation analysis, the correlation between the DANVA total score and the mPFC/ACC E/I ratio of the BD patients was no longer significant (*p* = 0.873). Hence, we proposed a model of the mediating effect of neurocognitive ability in emotion recognition. We tested the proposed model with the Sobel analysis (Figure 1) and found a significant mediating effect of neurocognition (WCST) on *E*/*I* balance in the mPFC/ACC and emotion recognition (*Z* = −2.657, *p* = 0. 007).

## Discussion

Building on our previous research demonstrating an association between E/I imbalance within the DMN—particularly in the PCC and mPFC/ACC—and executive function in euBD (Tseng et al., 2024), the current study extends this line of investigation into the social cognitive domain. Specifically, we examined whether the same *E*/*I* imbalance is also related to emotion recognition and whether this association is mediated by executive function. To the best of our knowledge, this mediation model has not been previously examined in bipolar disorder. Our findings offer preliminary evidence suggesting a potential neurocognitive pathway linking neurometabolic alterations with social cognitive functioning. To the extent of our knowledge, this research represents the first investigation into the correlation between *E*/*I* imbalance and emotion recognition in BD. We found that the *E*/*I* ratio in the mPFC/ACC negatively predicted emotion recognition ability in BD, the negative association was observed when the WCST categories completed were added to the model. The Sobel test proved that a mediating effect of cognitive flexibility on the correlation between the *E*/*I* ratio and emotion recognition in the mPFC/ACC. Consistent with the hypothesis, the results support the association between higher *E*/*I* balance within the DMN and poorer social cognition. Additionally, neurocognitive functioning, particularly executive function, was found to mediate this relationship.

Metabolite alterations were also mentioned in cross-sectional MRS studies comparing BD patients with HCs (Sosa-Moscoso et al., 2022), and the regions most commonly affected in BD include the prefrontal cortex, anterior cingulate cortex, and basal ganglion (Sosa-Moscoso et al., 2022). The neocortex is primarily composed of glutamatergic excitatory pyramidal neurons, making up the majority of its neuronal population (Somogyi, Tamás, Lujan, & Buhl, 1998). Approximately 20% of cortical neurons are GABAergic inhibitory interneurons (Tatti, Haley, Swanson, Tselha, & Maffei, 2017). The brain’s information processing relies on a delicate equilibrium between the excitatory signals generated by glutamatergic pyramidal neurons and the inhibitory signals from GABAergic interneurons. The interaction between the two contrasting processes of excitation and suppression can impact various neural circuits and brain function (Liu et al., 2021; Sohal & Rubenstein, 2019) and has been suggested as a framework for understanding the pathophysiology of neurodevelopmental and neuropsychiatric disorders (Liu et al., 2021).

The regional E/I balance has been shown to predict DMN function, which is associated with goal-directed task performance (Gu, Hu, Chen, He, & Yang, 2019). Effective suppression of DMN activity is essential for goal-directed cognition, potentially by reducing goal-irrelevant processes such as mind wandering. This framework aligns with our series of studies, in which we found that the *E*/*I* ratio in the mPFC/ACC negatively predicted both emotion recognition and executive function in individuals with BD (Tseng et al., 2024). These findings further support the notion that an optimal *E*/*I* balance in the mPFC/ACC is critical for regulating DMN function, thereby influencing cognitive and socio-emotional processes. Studies have found that individuals with schizophrenia and their siblings show impairments in both facial emotion recognition and executive functions, with correlations between these deficits (Jaracz, Grzechowiak, Raczkowiak, & Rybakowski, 2011; Yang et al., 2015). Similar findings were observed in BD patients (David, Soeiro-de-Souza, Moreno, & Bio, 2014). Higher emotional intelligence has been associated with better WCST performance, indicating a link between emotional competencies and prefrontal cortex functions (Alipour, Arefnasab, & Babamahmoodi, 2011). These findings underscore the interconnectedness of executive functions and emotional processing across different populations, aligning with the results of our own research. The mediating effect of neurocognition on emotional recognition was demonstrated through a model, where bottom-up perceptual processes are connected to social cognitive skills, such as facial recognition. These abilities are consistently influenced by neurocognitive functions, collectively contributing to the ability to recognize emotions (Ventura, Wood, Jimenez, & Hellemann, 2013). Extending our findings, we propose an explanation linking neurometabolic imbalance to impaired emotion recognition through E/I dysregulation in the PFC. Specifically, we suggest that an increased E/I ratio within the mPFC/ACC may disrupt the balance between excitatory and inhibitory signaling in cortical microcircuits—an equilibrium essential for neural decorrelation and efficient information processing (Chini, Pfeffer, & Hanganu-Opatz, 2022). This imbalance has been shown in animal studies to impair both cellular computation and social behavior (Yizhar et al., 2011), indicating functional consequences that span molecular, circuit, and behavioral levels. At the cognitive level, an elevated *E*/*I* ratio may interfere with evidence accumulation and decision-making, leading to impulsive judgments that overweight early cues (Lam et al., 2017). Computational modeling supports this notion, demonstrating that even subtle elevations in *E*/*I* ratio can destabilize decision dynamics and promote impulsive selections (Murray & Wang, 2018). Such effects are especially relevant to emotion recognition tasks, which require the dynamic integration of subtle facial or vocal cues over time. As Freeman et al. emphasize, person perception involves a continuous interplay between bottom-up and top-down information (Freeman, Johnson, Adams, & Ambady, 2012). Disruption of this temporal integration—potentially due to early sensory bias driven by *E*/*I* imbalance—may thus impair the accurate decoding of emotional signals (Freeman et al., 2012; Lam et al., 2017). Together, these findings provide a plausible mechanistic framework linking prefrontal neurometabolic imbalance to socio-cognitive dysfunction, mediated through impaired executive functioning.

There were some limitations of our study. First, the number of subjects included in the study was small, so caution is needed to generalize these findings to BD patients as a whole. Larger samples are needed to obtain more reliable results. Moreover, a potential limitation of this study is the lack of control for years of education, specifically in the within-group analyses. While education level is often considered a proxy for premorbid intellectual functioning and is known to influence performance on tasks of emotion recognition and executive function, its interpretation within clinical samples requires caution. In individuals with bipolar disorder, educational attainment may not solely reflect premorbid cognitive capacity, but also the cumulative impact of illness-related factors such as early onset, chronicity, or cognitive decline. As such, adjusting for education in analyses could lead to overadjustment and inadvertently remove variance that is meaningful for understanding illness-related neurobiological changes.

In the present study, our primary analyses were conducted without controlling for education in order to preserve the interpretability of illness-related variance. Nevertheless, to address potential confounding, we performed sensitivity analyses controlling for education, and these results—presented in the Supplementary Materials—showed attenuation of the correlation between *E*/*I* balance and emotion recognition.

Higher educational attainment has been implicated in cognitive resilience and structural brain modifications, including increased gray matter volume and metabolic activity in the anterior cingulate cortex, lingual gyri, and precuneus (Arenaza-Urquijo et al., 2013; Eisenberg et al., 2005). However, the relationship with neurometabolite concentrations remains inadequately characterized. While limited evidence suggests that greater educational attainment is associated with elevated whole-brain N-acetylaspartate levels—an established marker of neuronal integrity—particularly in younger adults (Glodzik et al., 2012), the precise nature and extent of this association remain unclear. Consequently, the years of education which was not controlled. Additionally, there are some subtype features and multiple dimensions within BD (Angst, 2007), and these may contribute to different neurometabolite characteristics. On the other hand, MRS measurement variability depends on many technical factors, including hardware, acquisition parameters, data quality, and data analysis (Harris et al., 2022). Additional biological challenges with MRS are that metabolite levels are linked to the tissue fraction in the voxel; moreover, neurometabolites may change with age (Harris et al., 2022). Concerning those MRS limitations, studies evaluating the roles of neurometabolites need careful interpretation and take MRS harmonization into consideration. Finally, several animal models have proven that mood stabilizers exert therapeutic effects by altering the synaptic *E*/*I* balance (Lee, Zhang, Kim, & Han, 2018), which may have confounded our results. Further comparison of subgroups, such as medicated and non-medicated BD, BDI/II patients, or patients with psychotic features or not, in a larger sample size is warranted in the future. Lastly, this cross-sectional study had limitations in identifying causal inference and establishing neurobiological pathways.

In summary, this study provides preliminary evidence for a possible mechanistic pathway in which *E*/*I* imbalance may influence social cognition indirectly through executive functioning, specifically affecting emotion recognition in euBD. This finding suggests that prefrontal executive function, particularly cognitive flexibility, could play a mediating role linking neurobiological dysregulation to social cognitive performance, addressing an area that has received limited attention in the literature.

These results may also have practical implications. They indicate that social cognitive interventions might be enhanced by including components that support executive functions, such as cognitive flexibility and working memory, in addition to conventional social scenario training. Such an integrated approach could potentially increase the effectiveness of psychosocial interventions and contribute to improved functional outcomes.

## Abbreviations

^1^H-MRS: Proton magnetic resonance spectroscopy
ANCOVA: analysis of covariance
CRLB: Cramér-Rao lower bound
DANVA-2-TW: Diagnostic Analysis of Nonverbal Accuracy 2-Taiwan version
DMN: default mode network
E/I: excitatory/inhibitory
euBD: Euthymic bipolar disorder
Glx: glutamate/glutamine
HCs: healthy controls
HDRS: Hamilton Depression Rating Scale
mPFC/ACC: medial prefrontal cortex/anterior cingulate cortex
PCC: posterior cingulate cortex
PRESS: point-resolved spectroscopy
WCST: Wisconsin Card Sorting Test
YMRS: Young Mania Rating Scale
